# Supplementary material for: Crystal structure of a multi-domain human smoothened receptor in complex with a super stabilizing ligand
Source: Nat Commun. 2017 May 17;8:15383. doi: 10.1038/ncomms15383 (PMC5442369; doi:10.1038/ncomms15383)
Supplement: Supplementary Information — Supplementary figures, supplementary table, supplementary methods and supplementary references. [file ncomms15383-s1.pdf]

a

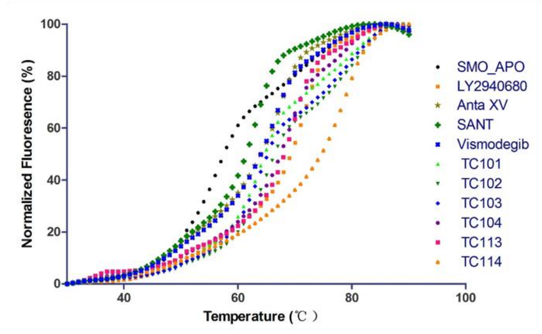

b

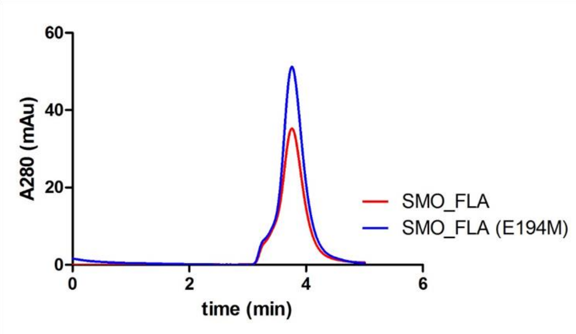

Supplementary Figure 1. Expression and stability assay of SMO-FLA constructs. a, CPM thermal shift assay[1] of SMO in an apo state (black) or incubated with 20 µM of different ligands. The melting temperature (T_m) of SMO supplemented with ligands is higher than that of the apo protein, which demonstrates that these ligands bind to the receptor and directly influence the behavior of SMO improving its thermostability. **b,** aSEC traces of SMO-FLA constructs to characterize the protein yield and homogeneity. The construct used for XFEL and synchrotron data collection are shown in blue and red traces, respectively.

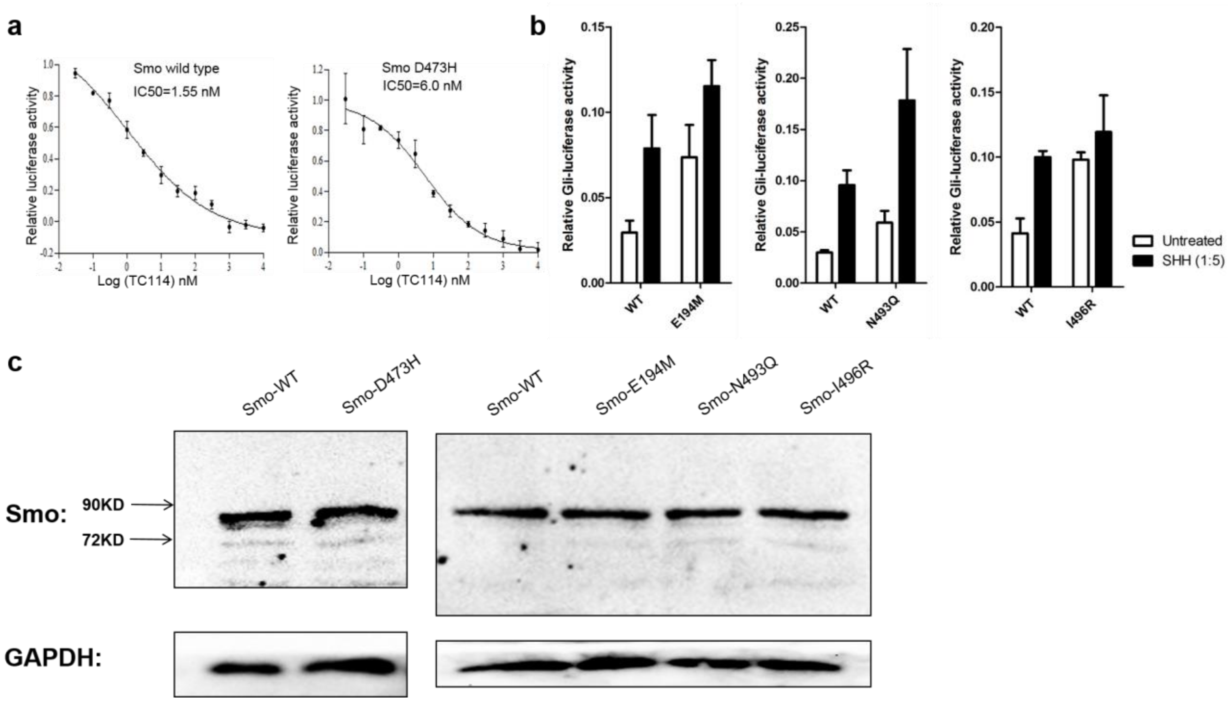

Supplementary Figure 2. Cell-based functional characterization for TC114 and SMO mutants. a, Antagonist effect of compound TC114 on the Gli-luciferase activity in light II cells transfected with SMO WT and drug resistant mutant D473H, and stimulated by SAG. **b,** Gli-luciferase activity in light II cells transfected with SMO WT and mutants, with or without exposure to Sonic Hedgehog (SHH). **c,** Expression level of SMO WT or mutants in light II cells was examined by western blot analysis. GAPDH was used as a loading control. Each luciferase data point was represented as mean ± SD repeated in triplicates. Data were analyzed using GraphPad software.

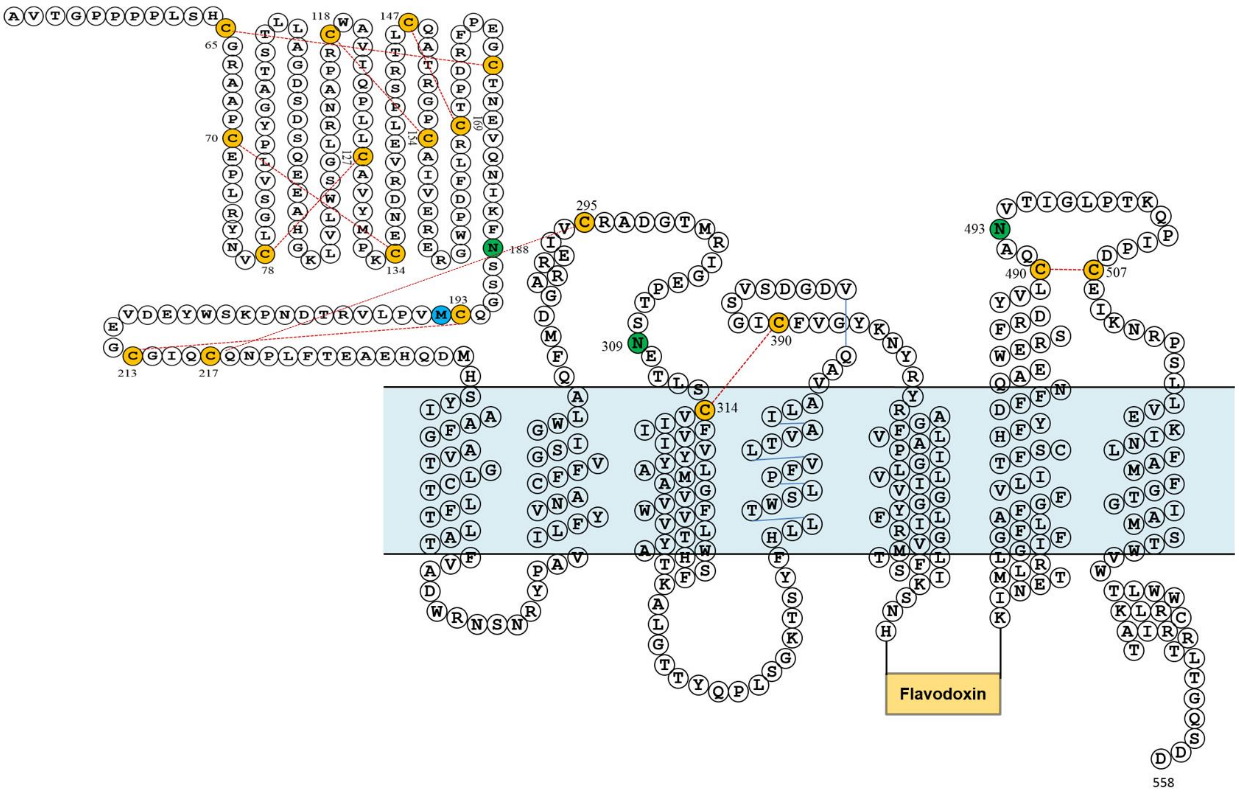

Supplementary Figure 3. Snake plot of crystallized SMO construct. In order to improve expression and increase the likelihood of crystallization, we modified the SMO receptor. An engineered construct of the human SMO contains a Flavodoxin fusion replacing part of ICL3 between P434 to S443. The N terminus 1-52 and C terminus 559-787 were truncated. The residue E194 colored in blue was mutated into M in the hinge domain (HD). In the snake plot representation, cysteines that form disulfide bonds are colored orange. The putative glycosylation sites are colored green. This construct was used for XFEL data collection. The construct for synchrotron data collection has the N-terminus truncated beyond P57 and lacks the E194M mutation.

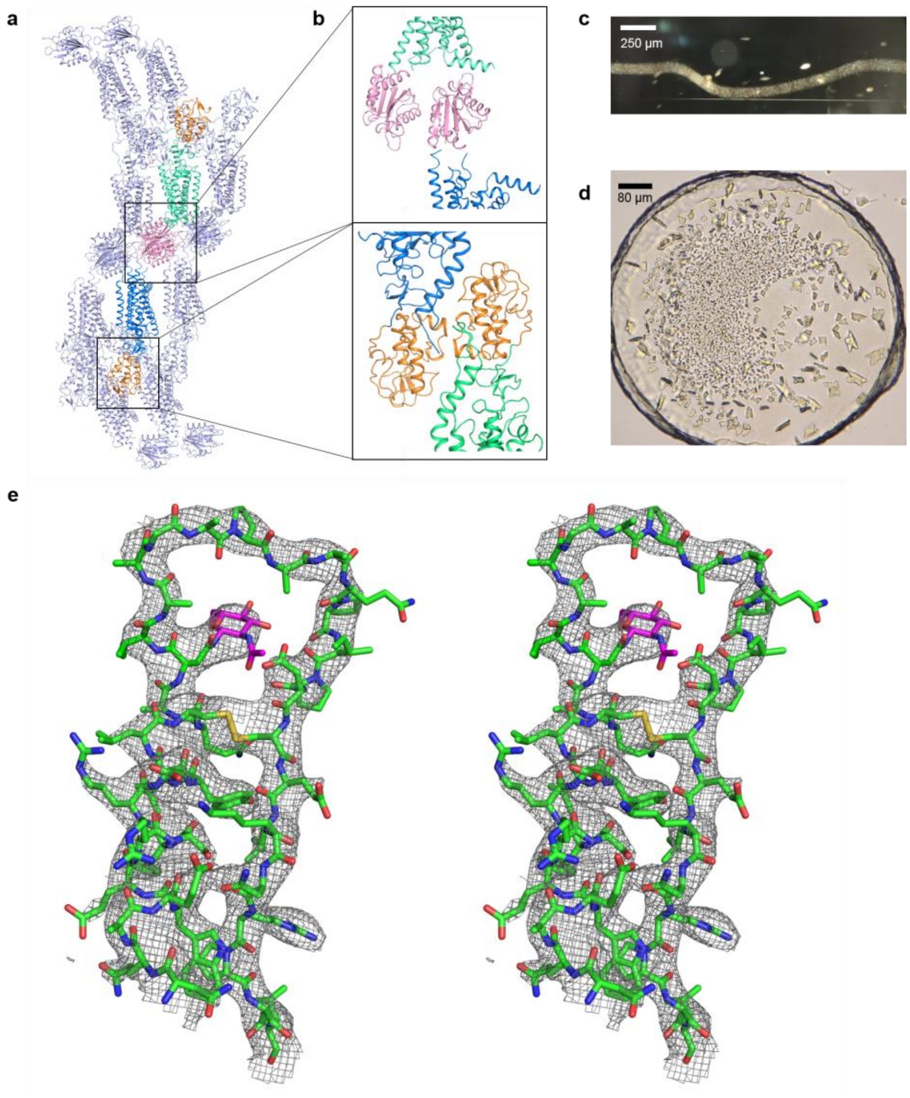

Supplementary Figure 4. Crystal packing and SMO_TC114 crystals. a, Crystal packing of the XFEL structure with protein molecules shown in cartoon presentation. One asymmetric unit contains two SMO-TC114 molecules packed tail-to-tail and colored in lime green and marine for TMD, orange for CRD and pink for Flavodoxin. **b,** Close-up view of crystal packing. **c, d,** Crystals of SMO_TC114 complex grown in a syringe for XFEL data collection and an LCP sandwich plate for synchrotron data collection, respectively. **e,** A stereo image of the ECL3 portion of the electron density map (|2Fo|-|Fc|, contour level at 1). Residues are shown in green sticks. Glycan moiety (NAG) is shown in magenta.

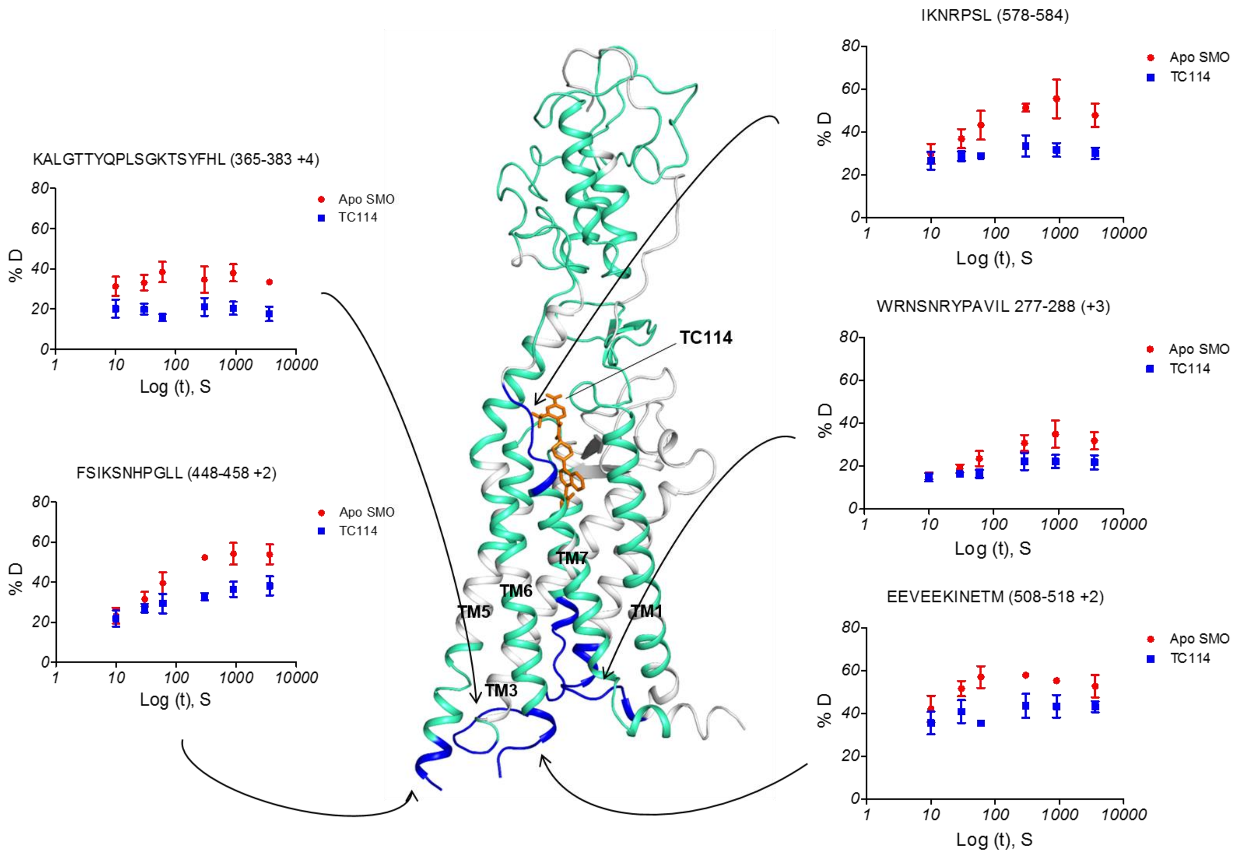

Supplementary Figure 5. Stabilization of SMO by TC114 in the HDX studies. Changes in the average percent deuterium uptake are mapped on the SMO crystal structure. Blue regions show areas of >5% decreased exchange in TC114-bound compared to apo receptor. Green cyan show regions with no significant difference of exchange (0-5%) between apo and TC114-bound states. White regions have no peptic peptides covering that sequence in MSMS searches and HDX runs. Representative D_2O uptake curves for peptides in the regions that are different between apo (red) and TC114-bound (blue) are shown around the structure. The data are shown as mean ± s.d. of three independent experiments.

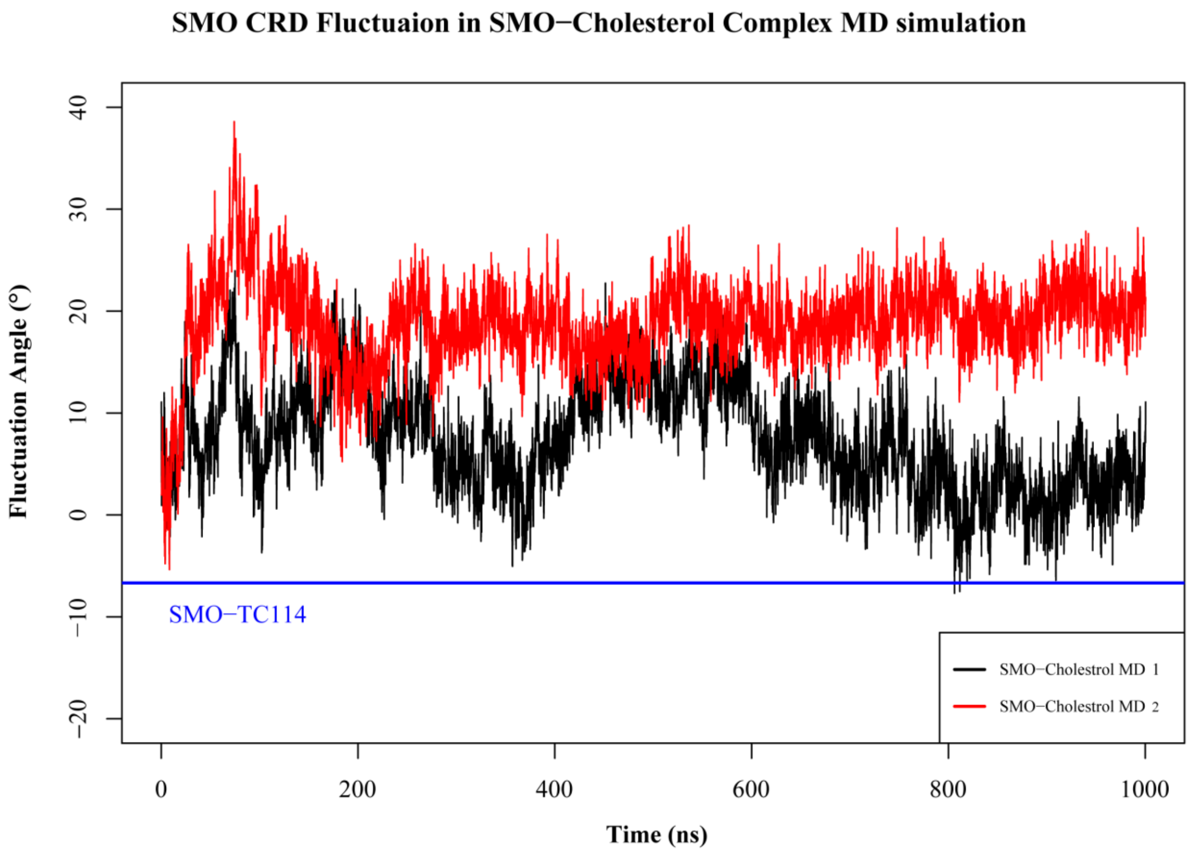

Supplementary Figure 6. The fluctuation of SMO CRD relative to SMO TMD from molecular dynamic simulation. The tilt angle of CRD-TMD was simply defined by 3 atoms (C-alpha of P69, V210, W535). The blue line indicates ~7° twist angle of SMO-TC114 relative to SMO-Cholesterol complex toward outward direction of the membrane plane. The black and red plots indicate two independent MD simulations respectively, and both of them show an opposite direction tilt of SMO-TC114. These two MD results indicate that SMO CRD tilts toward inward direction of the membrane plane rather than stay in the original 5L7D crystal state.

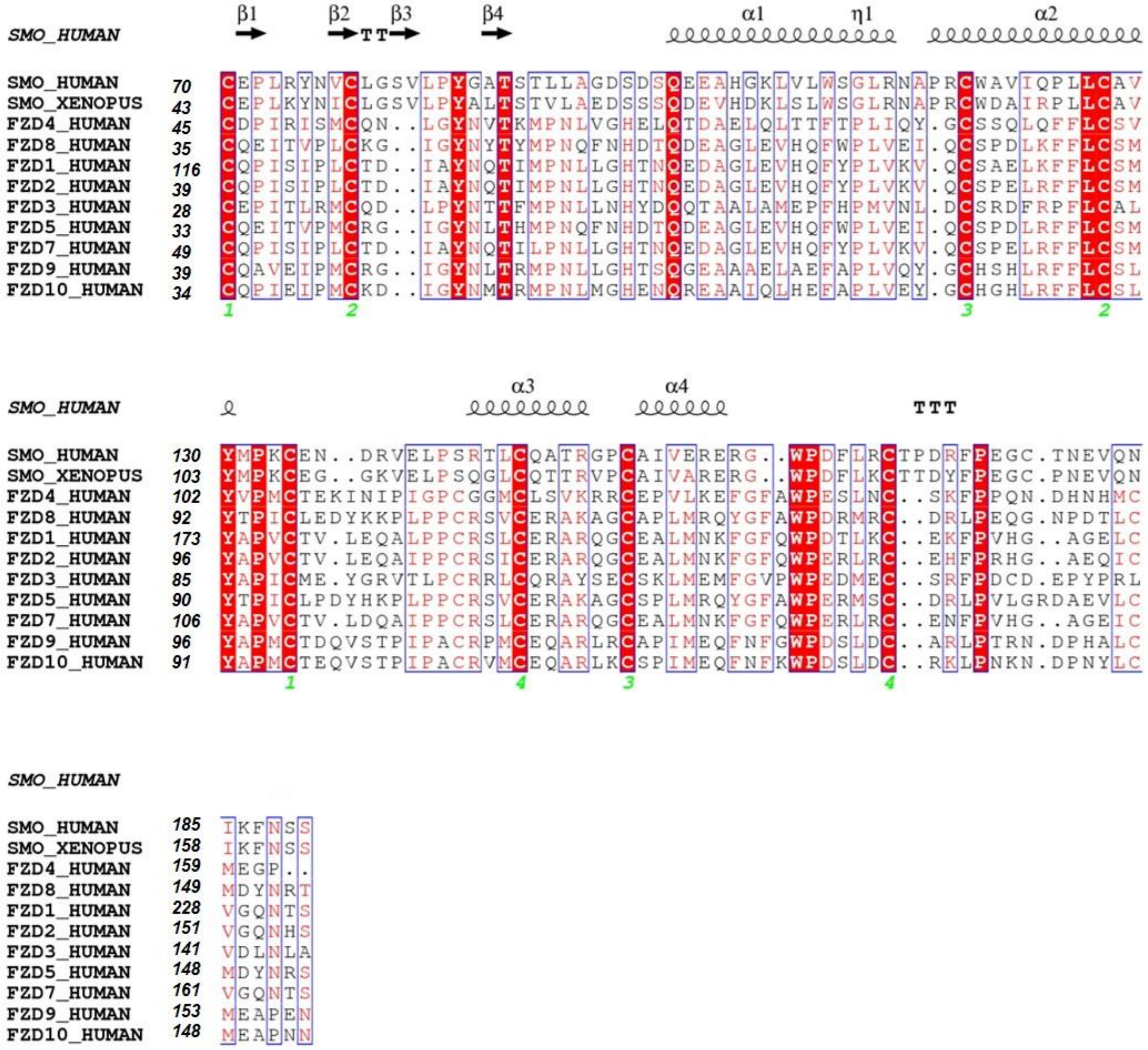

Supplementary Figure 7. CRD Sequence alignment between the SMO and FZD receptors. Secondary structural elements in human SMO CRD structure are shown on top of the sequences[2]. Four pairs of conserved disulfide bonds are highlighted with green numbers under the sequences. Fully conserved residues are shown as white letters on red background. Partially conserved residues are shown as red letters on white background.

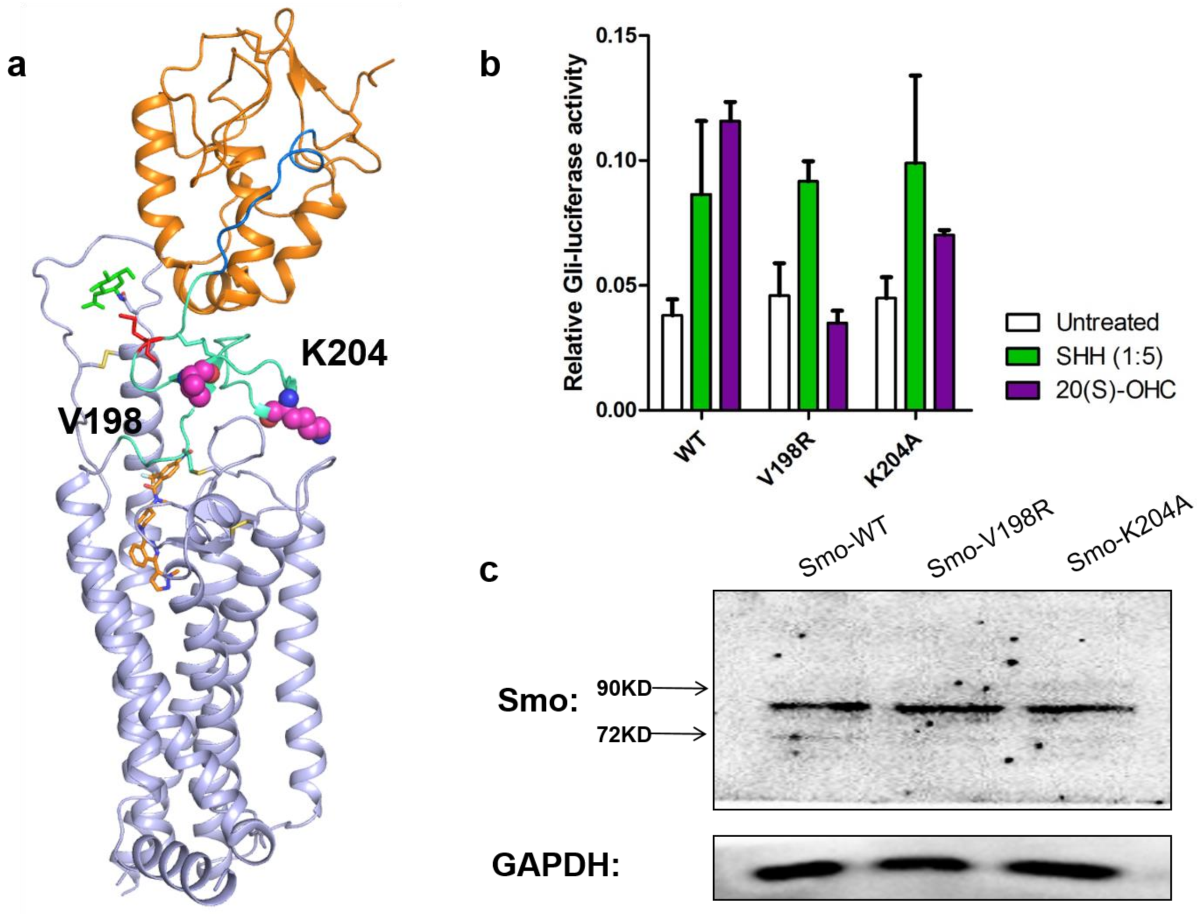

Supplementary Figure 8. Cell-based functional characterization for HD mutations. a, Structure of human SMO, with the CRD, linker, HD and TMD indicated as orange, marine, green cyan and light blue cartoons respectively. The two HD mutation sites are highlighted as magenta spheres. **b,** Gli-luciferase activity in light II cells transfected with SMO WT or mutants, and stimulated by Sonic Hedgehog (SHH) or 20(*S*)-OHC. **c,** Expression level of SMO WT or mutants in light II cells was examined by western blot analysis. GAPDH was used as a loading control. Each luciferase data point was represented as mean ± SD repeated in triplicates. Data were analyzed using GraphPad software.

Supplementary Table 1. Primer sequences used in this study.

ICL3-Fla-F	GACTCTGTTTAGCATTAAGAGCAATCATGCCAAGGCTCTCATCGTG TATGGAAGCA
ICL3-Fla-R	CCAGGCGCAGCATAGTTTCGTTAATTTTAATAGCGCCCCTCACGTC ATGGGCCCAG
D-N52 -F	CCTGTATTTTCAGGGCGCAGTGACTGGACCAC
D-N52 -R	GTGGTCCAGTCACTGCGCCCTGAAAATACAGG
C-Term-Truncation-F	TGGCCAGTCTGACGATCACCACCATCACCACC
C-Term-Truncation-R	GGTGGTGATGGTGGTGATCGTCAGACTGGCCA
E194M-F	TCTAGTGGGCAGTGCATGGTGCCTCTGGTCAGG
E194M-R	CCTGACCAGAGGCACCATGCACTGCCCACTAGA
D-N57 -F	CCTGTATTTTCAGGGCCCTCCACCACTGTCCC
D-N57 -R	GGGACAGTGGTGGAGGGCCCTGAAAATACAGG

Supplementary Methods

Synthesis of LY2940680 analogues

All reagents and solvents were obtained from commercial sources (Adamas, Energy Chemical and Sigma-Aldrich) and were used without further purification. Compound **2** was synthesized by following literature procedure[3]. Column chromatography was performed on silica gel 200-300 mesh. NMR spectra were recorded on a Bruker AVANCE III 500 spectrometer (FT, 500 MHz for ^{1}H NMR; 125 MHz for ^{13}C NMR) at room temperature with $CDCl_3$ as the solvent and tetramethylsilane (TMS) as the internal standard. Chemical shifts were reported in units (ppm) by assigning TMS resonance in the ^{1}H spectrum as 0.00 ppm and $CDCl_3$ resonance in the ^{13}C spectrum as 77.0 ppm. All coupling constants (*J* values) were reported in Hertz (Hz). High-resolution mass spectra (HRMS) were recorded on an Agilent 6230 mass spectrometer using ESI (electrospray ionization).

General Synthesis Scheme of LY2940680 analogues

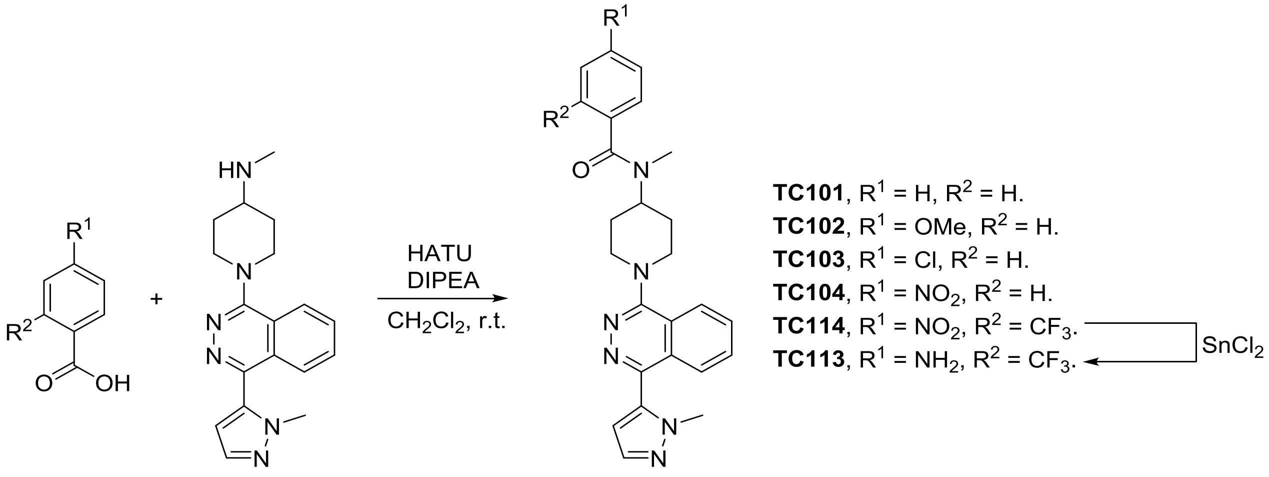

1 **2**

To a solution of benzoic acid **1** (0.34 mmol) in 3 mL CH_2Cl_2, secondary amine **2** (100 mg, 0.31 mmol) and *N,N*-diisopropylethylamine (DIPEA, 60 mg, 80 μL, 0.46 mmol) were added followed

by the treatment of 1-[bis(dimethylamino)methylene]-1*H*-1,2,3-triazolo[4,5-b]pyridinium 3-oxid hexafluorophosphate (HATU, 153 mg, 0.40 mmol). The reaction mixture was stirred at room temperature for 1 hour before being quenched by the addition of brine. The reaction mixture was extracted 3 times with CH_2Cl_2. The combined organic layer was washed with saturated $NaHCO_3$ solution and brine sequentially, dried over Na_2SO_4. After filtration, the solution was concentrated in vacuum and the crude product was purified by flash column chromatography on silica gel to yield the corresponding amide. For **TC104** and **TC114**, NMR showed a mixture of amide rotamers.

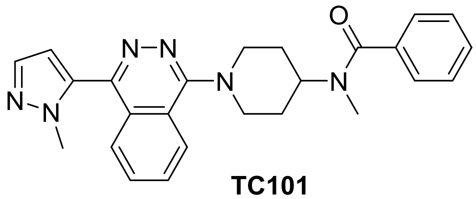

TC101

TC101: Colorless solid, isolated yield 62% (82 mg). ^{1}H NMR (500 MHz, $CDCl_3$), δ (ppm) 1.87-2.21 (m, 4H, CH_2), 2.93 (s, 3H, CH_3), 2.98 (br, 1H, CH_2), 3.38 (br, 1H, CH_2), 4.05 (s, 4H, CH_3 and CH_2), 4.19 (br, 1H, CH_2), 4.90 (br, 1H, CH), 6.59 (s, 1H, CH), 7.43 (br, 5H, CH), 7.66 (d, J = 1.5 Hz, 1H, CH), 7.81-7.90 (m, 2H, CH), 8.06-8.10 (m, 2H, CH); ^{13}C NMR (125 MHz, $CDCl_3$), δ 17.6, 29.5, 29.6, 30.6, 38.2, 49.3, 50.7, 109.1, 121.4, 124.6, 126.2, 127.9, 128.5, 129.5, 131.5, 131.9, 136.7, 136.8, 138.1, 159.4; HRMS calcd for $C_{25}H_{26}N_6O$ $[M+H]^+$: 427.2247; found: 427.2245.

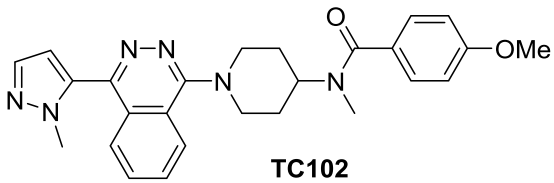

TC102

TC102: Colorless solid, isolated yield 60% (85 mg). ^{1}H NMR (500 MHz, $CDCl_3$) δ (ppm) 1.95-2.22 (m, 4H, CH_2), 3.01-3.36 (m, 5H, CH_3 and CH_2), 3.85 (s, 3H, CH_3), 4.05-4.19 (m, 5H, CH_3 and CH_2), 4.84 (br, 1H, CH), 6.59 (d, J = 1.5 Hz, 1H, CH), 6.94 (d, J = 8.5 Hz, 2H, CH), 7.42 (d, J = 8.5 Hz, 2H, CH), 7.66 (d, J = 1.5 Hz, 1H, CH), 7.82-7.90 (m, 2H, CH), 8.06 (d, J = 8.5 Hz, 1H, CH), 8.11 (d, J = 8.0 Hz, 1H, CH); ^{13}C NMR (125 MHz, $CDCl_3$), δ 22.6, 25.4, 27.1, 31.8, 38.2, 38.5, 50.6, 55.2, 109.0, 113.6, 121.3, 124.6, 126.1, 127.8, 128.8, 131.4, 131.9, 136.7, 138.1, 159.4, 160.5; HRMS calcd for $C_{26}H_{28}N_6O_2$ $[M+H]^+$: 457.2347; found: 457.2340.

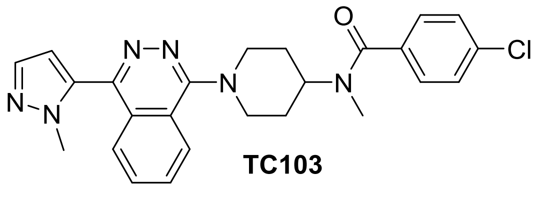

TC103

TC103: Colorless solid, isolated yield 55% (78 mg). ^{1}H NMR (500 MHz, $CDCl_3$), δ (ppm) 1.83-2.20 (m, 4H, CH_2), 2.93 (s, 3H, CH_3), 3.08 (br, 1H, CH_2), 3.40 (br, 1H, CH_2), 4.06 (s, 4H, CH_3 and CH_2), 4.19 (br, 1H, CH_2), 4.86 (br, 1H, CH), 6.59 (d, J = 2.0 Hz, 1H, CH), 7.37-7.43 (m 4H, CH), 7.66 (d, J = 2.0 Hz, 1H, CH), 7.82-7.90 (m, 2H, CH), 8.06-8.10 (m, 2H, CH); ^{13}C NMR (125 MHz, $CDCl_3$), δ 28.6, 29.6, 32.5, 38.3, 50.57, 50.63, 109.1, 121.3, 124.6, 126.2, 127.9, 128.4, 128.8, 131.5, 132.0, 135.1, 135.6, 136.7, 138.1, 147.5, 159.4; HRMS calcd for $C_{25}H_{25}ClN_6O$ $[M+H]^+$: 461.1851; found: 461.1848.

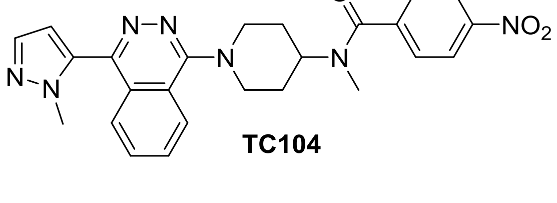

TC104

TC104: Colorless solid, isolated yield 47% (69 mg). ^{1}H NMR (500 MHz, $CDCl_3$) major rotamer, δ (ppm) 1.84-2.33 (m, 4H, CH_2), 2.93 (s, 3H, CH_3), 3.39-3.44 (m, 2H, CH_2), 4.07 (s, 3H, CH_3), 4.22-4.25 (m, 2H, CH_2), 4.88-4.93 (m, 1H, CH), 6.60 (br, 1H, CH), 7.61-7.66 (m, 3H, CH), 7.84-7.92 (m,2H, CH), 8.07-8.15 (m, 2H, CH), 8.30-8.32 (m, 2H, CH); minor rotamer, δ (ppm) 1.84-2.33 (m, 4H, CH_2), 3.13 (s, 3H, CH_3), 2.90-3.04 (m, 2H, CH_2), 3.66-3.73 (m, 2H, CH_2), 4.03 (s, 3H, CH_3), 5.34-5.36 (m, 1H, CH), 6.60 (br, 1H, CH), 7.61-7.66 (m, 3H, CH), 7.84-7.92 (m,2H, CH), 8.07-8.15 (m, 2H, CH), 8.30-8.32 (m, 2H, CH); ^{13}C NMR (125 MHz, $CDCl_3$) major rotamer, δ 27.0, 28.4, 29.7, 32.2, 38.1, 50.5, 51.6, 109.0, 121.2, 123.7, 126.1, 127.78, 127.80, 131.4, 131.9, 136.6, 138.0, 142.8, 147.3, 148.0, 159.3, 165.6, 169.1; minor rotamer, δ (ppm) 22.5, 25.4, 27.8, 31.7, 38.1, 50.2, 56.9, 107.6, 124.0, 124.5, 127.1, 128.3, 128.4, 131.6, 132.0, 136.5, 138.0, 142.8, 147.6, 148.0, 159.1, 165.6, 169.4; HRMS calcd for $C_{25}H_{25}N_7O_3$ $[M+H]^+$: 472.2092; found: 472.2087.

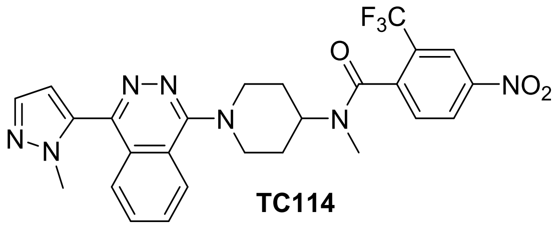

TC114

TC114: Colorless solid, isolated yield 49% (82 mg). ^{1}H NMR (500 MHz, $CDCl_3$) major rotamer, δ (ppm) 1.84-2.36 (m, 4H, CH_2), 2.77 (s, 3H, CH_3), 3.60-3.67 (m, 2H, CH_2), 4.01 (s, 3H, CH_3), 4.39-4.46 (m, 2H, CH_2), 4.88-4.93 (m, 1H, CH), 6.65 (d, J = 1.5 Hz, 1H, CH), 7.62 (d, J = 8.5 Hz, 1H, CH), 7.70 (d, J = 1.5 Hz, 1H, CH), 7.98-8.06 (m, 2H, CH), 8.09-8.15 (m, 1H, CH), 8.25-8.27 (m, 1H, CH), 8.49-8.52 (m, 1H, CH), 8.60 (d, J = 1.5 Hz, 1H, CH); minor rotamer, δ (ppm) 1.84-2.36 (m, 4H, CH_2), 3.14 (s, 3H, CH_3), 3.12-3.21 (m, 2H, CH_2), 3.45-3.49 (m, 1H, CH), 3.98

(s, 3H, CH_3), 4.18-4.20 (m, 2H, CH_2), 6.63 (d, J = 1.5 Hz, 1H, CH), 7.61 (d, J = 8.5 Hz, 1H, CH), 7.69 (d, J = 1.5 Hz, 1H, CH), 7.98-8.06 (m, 2H, CH), 8.09-8.15 (m, 1H, CH), 8.25-8.27 (m, 1H, CH), 8.49-8.52 (m, 1H, CH), 8.60 (d, J = 1.5 Hz, 1H, CH); ^{13}C NMR (125 MHz, $CDCl_3$) major rotamer, δ 27.8, 28.2, 32.0, 38.1, 50.7, 51.3, 51.5, 109.9, 122.2, 122.42 (q, J = 272.9 Hz), 122.45 (q, J = 4.7 Hz), 126.2, 127.3, 128.2 (q, J = 33.5 Hz), 128.83, 128,85, 133.0, 134.1, 134.6, 138.3, 141.1 (q, J = 2.8 Hz), 146.3, 147.7, 157.4, 166.8; minor rotamer, δ (ppm) 27.7, 28.9, 29.3, 38.0, 50.1, 51.0, 56.3, 109.8, 115.6 (q, J = 286.1 Hz), 122.0, 122.8 (q, J = 4.7 Hz), 125.5, 127.1, 128.39, 128.42 (q, J = 33.5 Hz), 128.6, 128.8, 132.9, 134.6, 135.0, 138.2, 140.8 (q, J = 2.3 Hz), 146.8, 147.8, 158.0, 166.5; HRMS calcd for $C_{26}H_{24}F_3N_7O_3$ $[M+H]^+$: 540.1965; found: 540.1964.

Synthesis of **TC113**

To a solution of **TC114** (167mg, 0.31 mmol) in 3 mL ethyl acetate, a solution of $SnCl_2$ (586 mg, 3.1 mmol) in 3 mL H_2O was added. The reaction was stirred for 12 hour at room temperature before being quenched by the addition of 1M NaOH solution. The reaction mixture was extracted 3 times with ethyl acetate. The combined organic layer was dried over Na_2SO_4. After filtration, the solution was concentrated in vacuum and the crude product was purified by flash column chromatography on silica gel to yield **TC113**. NMR showed a mixture of amide rotamers.

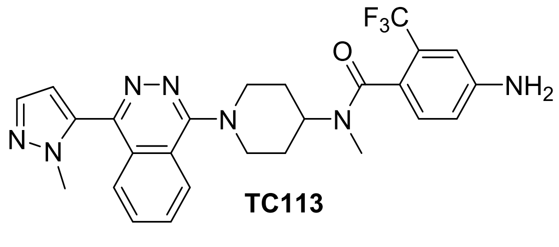

TC113

TC113: Colorless solid, isolated yield 52% (82 mg). ^{1}H NMR (500 MHz, $CDCl_3$) major rotamer, δ (ppm) 1.76-2.24 (m, 4H, CH_2), 2.78 (s, 3H, CH_3), 3.34-3.40 (m, 2H, CH_2), 4.06 (s, 3H, CH_3),

4.18-4.23 (m, 4H, CH_2), 4.90-4.94 (m, 1H, CH), 6.60 (d, J = 1.5 Hz, 1H, CH), 6.83 (d, J = 8.0 Hz, 1H, CH), 6.94 (d, J = 1.5 Hz, 1H, CH), 7.09 (d, J = 8.0 Hz, 1H, CH), 7.66 (d, J = 2.0 Hz, 1H, CH), 7.81-7.90 (m, 2H, CH), 8.03-8.13 (m, 2H, CH); minor rotamer, δ (ppm) 1.76-2.24 (m, 4H, CH_2), 3.09 (s, 3H, CH_3), 2.94-3.09 (m, 2H, CH_2), 3.57-3.62 (m, 1H, CH), 4.02 (s, 3H, CH_3), 4.11-4.18 (m, 4H, CH_2), 6.59 (d, J = 1.5 Hz, 1H, CH), 6.83 (d, J = 8.0 Hz, 1H, CH), 6.96 (d, J = 1.5 Hz, 1H, CH), 7.09 (d, J = 8.0 Hz, 1H, CH), 7.65 (d, J = 2.0 Hz, 1H, CH), 7.81-7.90 (m, 2H, CH), 8.03-8.13 (m, 2H, CH); ^{13}C NMR (125 MHz, $CDCl_3$) major rotamer, δ 17.5, 27.5, 31.7, 38.11, 38.16, 51.0, 56.7, 109.0, 112.0 (q, J = 4.5 Hz), 117.6, 121.3, 123.6 (q, J = 272.2 Hz), 124.6, 124.8 (q, J = 2.5 Hz), 126.0, 127.4 (q, J = 31.4 Hz), 127.8, 128.2, 131.4, 131.9, 136.7, 138.0, 147.2, 147.3, 159.5, 169.6; minor rotamer, δ (ppm) 14.1, 29.1, 29.5, 30.6, 49.3, 50.1, 50.9, 109.8, 112.2 (q, J = 4.5 Hz), 117.4, 121.3, 123.6 (q, J = 272.2 Hz), 124.0 (q, J = 2.1 Hz), 124.4, 126.1, 127.4 (q, J = 31.4 Hz), 127.78, 127.80, 131.6, 132.0, 136.6, 138.1, 147.4, 147.6, 159.4, 169.6; HRMS calcd for $C_{26}H_{26q}F_3N_7O$ $[M+H]^+$: 510.2224; found: 510.2221.

Supplementary References:

1. Alexandrov, A.I., Mileni, M., Chien, E.Y., Hanson, M.A. & Stevens, R.C. Microscale fluorescent thermal stability assay for membrane proteins. *Structure* **16**, 351-359 (2008).
2. Robert, X. & Gouet, P. Deciphering key features in protein structures with the new ENDscript server. *Nucleic acids research* **42**, W320-324 (2014).
3. Hipskind, P.A., Patel, B.K. & Wilson, T. Disubstituted phthalazine hedgehog pathway antagonists. US Patent 8,273,742 (2012).
